# The value of domiciliary medication reviews – a thematic analysis of pharmacist’s views

**DOI:** 10.1007/s11096-022-01427-2

**Published:** 2022-07-14

**Authors:** Patricia McCormick, Bridget Coleman, Ian Bates

**Affiliations:** 1 School of Pharmacy, University College London, 29-39 Brunswick Square, Bloomsbury, WC1N 1AX London, England; 2 Level 1, Pharmacy Department, Whittington Health, Whittington Hospital, Magdala Avenue, N19 5NF London, England

**Keywords:** Medication review, Home care services, Pharmacists, Outcome Assessment, Health Care, Evidence-Based Pharmacy, Practice, Qualitative Research

## Abstract

**Background:**

Domiciliary medication reviews are thought to enable comprehensive medication reviews centred around the needs of individuals. However, there is no clear consensus on where the value of these services lie.

**Aim:**

To determine the value of domiciliary medication reviews to service providers through semi-structured focus groups, interviews and thematic analysis.

**Method:**

Study participants were recruited from domiciliary medication review services provided in the United Kingdom. Semi-structured focus groups and interviews were analysed using thematic analysis.

**Results:**

Six themes were identified: the scope of domiciliary medication review services, the professional role, advantages over traditional settings, disadvantages of domiciliary medication reviews for the professional, levels of engagement and outcomes.

**Conclusion:**

Pharmacy professionals believe that the domiciliary setting provides advantages over traditional healthcare settings when conducting medication reviews. They believe it enables a more in-depth review of an individual’s medications and needs. The traditional clinical outcomes recorded by services may not be capturing the holistic impact of domiciliary medication reviews.

## Impact statements

- This research represents an opportunity for DMR pharmacists to be recognised as advanced practitioners who can manage the complex needs of an individual.
- Results of the study suggest that the outcomes collated by services may not demonstrate the true value of DMR service, the patient perspective is missing. A move away from focusing on traditional clinical outcomes may be warranted.
- Components for success and barriers to delivery of DMR services have been identified which should be considered when planning service delivery.

## Introduction

Medication reviews involve the structured review of medications with the aim of optimising treatment to get the best possible outcomes [1]. Traditionally medication reviews have taken place in various healthcare settings such as community pharmacies, hospitals and General Practice surgeries [2]. In recent years, medication reviews in the domiciliary setting have become more prevalent [3]. Individuals residing in their homes commonly experience medicine related problems (MRPs) linked to polypharmacy and overall health complexity [4]. Domiciliary medication reviews are thought to enable comprehensive medication reviews centred around the needs of individuals. Research into DMRs has suggested that the impact of DMRs can be demonstrated by traditional clinical outcomes [5–8]. However, there is no clear consensus on where the value of these services lie.

An earlier study conducted by the authors examined where the users of domiciliary medication review services; patients felt the value of these services lay. The study found that domiciliary medication review were preferred to and provided advantages over traditional healthcare settings. DMRs were not time pressured, individuals felt they could discuss their objectives and that DMR professionals listened to them [9].

There have been studies examining the perspectives of pharmacists conducting medication reviews in primary care [10, 11] and community pharmacy settings [12–15]. These studies have focused on service delivery aspects of the reviews such as gaining patient engagement. There have been limited professional-focused qualitative studies focusing on the domiciliary setting for medication reviews [10, 16]. Studies have been process focused and do not delve into the global value of DMR services. There remains a gap in knowledge around where the pharmacy professionals who conduct domiciliary medication reviews feel the value of the services lies.

## Aim

To determine the value of domiciliary medication reviews to service providers through semi-structured focus groups, interviews and thematic analysis.

## Ethics approval

The study was sponsored by University College London. Ethical approval was granted (18/NI/0049) by the Office for Research Ethics Committees Northern Ireland (ORECNI) in March 2018. Health Research Authority (HRA) approval (IRAS: 232128) was also granted before the study took place. Study participants were assured their identity and any information provided would be kept anonymised. Informed consent was taken. Participation was voluntary and subjects were free to withdraw at any time.

## Method

Study participants were recruited from DMR services provided in the United Kingdom.

To identify potential DMR professionals an email that explained the research was circulated to authors of DMR services identified through an earlier systematic review conducted by the researcher. In addition a call for participants was posted on the websites of two professional networks: The Royal Pharmaceutical Society (RPS) the United Kingdom Clinical Pharmacy Association (UKCPA). Professionals were eligible to participate in the study if they were a current provider of a DMR service, or they had experience of delivering DMRs in the previous two years. A time limit on experience was included to limit potential recall bias.

Both focus groups and interviews were conducted in order to maximise practical issues regarding work scheduling for the respondents. The topic guide focused on six areas:

1. The professional’s background.
2. The origins of DMRs.
3. The professional’s expectations of the DMR.
4. The domiciliary setting.
5. Metrics and outcomes recorded for DMRs.
6. The professional’s view on what changes as a result of DMRs.

## Data collection and analysis

Twelve pharmacists participated in the study. Data collection took place between May and September 2018. Data collection was conducted by the primary researcher (PM). This study was the second qualitative study the researcher had undertaken. Prior to conducting the study training in qualitative data collection and analysis methods provided by the sponsoring institution was carried out. Audio files were transcribed into anonymised transcripts. The Braun and Clarke methodology for thematic analysis was followed [17]. NVivo® 11 software was used to manage the data. Data analysis began during the data collection period, through immersion in interview data. Each transcript was then coded. Codes were used to construct overarching themes from the data. Codes and emerging themes from each transcript were compared to existing findings within the data, using to a constant comparison approach [18, 19]. Initially the codes from earlier servicer user interviews were used as a codebook [9]. However, where codes did not fit with the data they were excluded, and emergent codes were added. The PhD supervisor (BC) read and coded 3 transcripts independently. All codes and themes were reviewed by both researchers (PM and BC) to ensure no duplication or ambiguity in meanings. Both researchers felt that data saturation had been reached by pharmacist ten.

## Results

All the professionals who participated in the study were pharmacists. Three focus groups took place first between May and August 2018. One took place virtually (P001 and P002) and two took place face-to-face. The first involved three participants (P003, P004 and P005) and the second involved two participants (P006 and P007). Five individual interviews were conducted via telephone for pharmacists who could not attend focus groups in August and September 2018. Interviews and focus groups varied in length from 16 to 41 min (Table 1) Five of the twelve participants were known to the researcher prior to participation in the study.

**Table 1:** Duration of focus groups and interviews

	Participants	Duration
Focus group 1	001 & 002	40 min
Focus group 2	003, 004 & 005	41 min
Focus group 3	006 & 007	31 min
Interview 1	008	41 min
Interview 2	009	16 min
Interview 3	010	21 min
Interview 4	011	32 min
Interview 5	012	22 min

Analysis of the data revealed six key themes and 23 sub-themes (Table 2). Illustrative quotes are used to demonstrate the meaning within themes.

**Table 2:** Themes and sub-themes from focus groups and interviews

Theme 1: The scope of DMRs	Theme 2: The professional role	Theme 3: Advantages over traditional settings	Theme 4: Disadvantages of DMRs for the professional	Theme 5: Levels of engagement	Theme 6: Outcomes
DMR stakeholders	Expanding professional boundaries	Mobility need	Time taken	Origins of DMR	Access outcomes
Pathways	Professional reward	Time spent	Safety	Individual objectives	Adherence outcomes
DMR process	Professional isolation	Comprehensiveness		Shared decision making	Clinical outcomes
		Inter-professional differences		Personability	Economic outcomes
					Humanistic outcomes
					Ideal world outcomes

## Theme 1: the scope of the DMRs

Opening questions covered the genesis and operational aspects of their DMR services.

### DMR stakeholders

The majority of DMR pharmacists interviewed worked within larger multi-professional teams who have a remit to support patients to remain independent in their home. Regardless, of where the DMR professional ‘sits’ they usually had interactions with a range of professionals from health and social care teams to meet the wide needs of patients. In addition, most of the pharmacists needed to communicate with GPs to discuss recommendations from the DMR. Interviewees highlighted that accessing GPs for these conversations was not easy, which presented a barrier in the DMR process.

### Pathways

The DMR services were presented as novel services. Some services, which had slightly different remits considered how they could work best with an existing service to enhance effectiveness.

### The DMR process

From the descriptions given it was clear that how DMRs were carried out across services, including what information systems they had access to, was different. Most pharmacists interviewed conducted broad medication reviews, i.e. not disease or condition specific. There was one exception, a pharmacist who focused on medication related issues linked to respiratory conditions. The need to complete some pre-review investigative work, to enable an effective review was highlighted in all focus groups and interviews.

## Theme 2: the professional role

Interviewees described their role as a DMR practitioner, how their professional boundaries had been stretched, the reward they experienced, and at times, the isolation they felt.

### Expanding professional boundaries

Professionals highlighted that they were completing tasks that are not traditionally considered ‘pharmacist’ roles. They had varying opinions on whether this was an appropriate use of their time or not. This was mainly discussed within the second focus group.

*It’s good, I would say it’s good but you find that you’re doing other peoples’ job and you’re not doing your own job. Because my medication reviews are behind and no one can do my job. [FG2: Pharmacist 003]*

Only one pharmacist, an independent prescriber, highlighted that they made the changes that they recommended themselves, without consulting another health care professional.

### Professional reward

Four pharmacists highlighted that they obtained a reward from carrying out interventions to meet an individuals’ medication related needs.

### Professional isolation

Some pharmacists highlighted that conducting DMRs left them professionally isolated, as generally they conducted reviews alone. In addition, whatever they discovered during a DMR they have to resolve themselves, which caused stress. The latter point was mainly discussed in one focus group.

*One drawback would be you’re kind of on your own and every now and then you come across things that you don’t feel comfortable to leave and it tends to be on a Friday afternoon and you’re still there at 8pm at night. (FG2: Pharmacist 003)*

## Theme 3: advantages over traditional settings

The pharmacists interviewed felt that DMRs provided advantages for the patient. The benefit of the DMR was felt in four key areas: mobility need, time spent, comprehensiveness and inter-professional differences.

### Mobility need

One pharmacist highlighted the advantages to those who might otherwise struggle to engage with professionals because of mobility issues:

*They don’t want to get on a bus or struggle waiting in a waiting room or anything. They prefer it. (FG2: Pharmacist 005)*

### Time spent

The majority of pharmacists reported that they spent more time with individuals conducting DMRs than they had in other settings. Professionals felt this time meant that they were able to conduct more meaningful reviews.

*The time factor really allows us to dig deep and check that everything is still appropriate and also check patient understanding. (FG3: Pharmacist 006)*

Pharmacists felt that individuals were also appreciative of the additional time spent with them.

*Most of the times the patients say this is the first time I’ve had someone to come in to talk to me about my medications for such a long time, to go through everything. (INT5: Pharmacist 012)*

### Comprehensiveness

The majority of pharmacists highlighted the comprehensive nature of DMRs. They reported that they encounter and try and resolve issues that were not identified from the outset i.e. issues not linked to the referral reason, and which at times, were not linked to medication related needs. They also highlighted that they could get a better understanding of an individuals needs in their own home than other care settings.

*The advantage of doing it in the home setting, is that you truly see what it’s like. And how they are coping with medicines so if you can just physically see a stack of blister packs dated a year back, you just know that they’re not coping. And it’s not something that you’d seen in hospital because the ambulance crew might just bring one blister pack from a pile of them. (FG3: Pharmacist 006)*

### Inter-professional differences

The pharmacists frequently highlighted how the home setting presented an opportunity for them to pick up on and resolve issues that other professionals had not picked up on. Various reasons were suggested for the limitations of other professionals. These included: not having the correct medication expertise, not having access to patient information systems and not having sufficient time to uncover and/ or resolve the problems.

*You’ve maybe dealt with some problems that kind of fell through the net so where the GP wouldn’t have had the time to maybe review all this person’s medications or wouldn’t have the incentive to do it... There is definitely a gap that you are filling, that no other professional is. (FG2: Pharmacist 005)*

There were examples of other health care professionals making recommendations to solve medication related problems that perhaps were not appropriate for the underlying problem.

*It was quite good yesterday for this Allied Health Care Professional, she’s an OT and one of the main drivers of “can we get a medibox? “(…) it was interesting for her to see that the patient there did have a medibox, there’s 6 sitting in the house and not a single medicine had been taken. (INT1: Pharmacist 008)*

## Theme 4: disadvantages of DMRs for the professional

Drawbacks to DMRs were discussed. Drawbacks to DMRs were not highlighted by every professional.

### Time taken

Some pharmacists expressed frustration at how much time it could take to get the problems they identified resolved. At times this was because they relied on other professionals who worked separately to the DMR pharmacist, to action requests.

### Safety

For a number of pharmacists, concerns around their personal safety, when going into someone’s home was a drawback to DMRs. They did not think about personal safety as much when they worked in other healthcare settings.

*There’s always a bit of nervousness because you’re not sure what the setting is going to be like and I guess you are aware of your own safety as well. (FG3: Pharmacist 007)*

## Theme 5: levels of engagement

When discussing how much patients participated in the DMR process, the pharmacists described three linked factors: individual objectives, shared decision making and the personability of the professional conducting the DMR.

### Origins of DMRs

All the pharmacists stated that DMRs came about after referral from another healthcare professional. Despite referrals originating from professionals rather than individuals, the pharmacists reported that negative responses from individuals when they were contacted to arrange the reviews were rare.

### Individual objectives

When a DMR was arranged individuals did not always have their own goals or issues to discuss. However, the pharmacists viewed part of their role as helping to elicit individual goals. The goals identified were not always linked to medications.

*It is very much driven by them, you know what is going on, what about your medicines, and then what they show you, sometimes it is horrifying, the cupboards full of medicines, I’ve taken away bags and bags of medicines and sometimes that’s a great relief for patients because they’ve obviously been building up and overwhelming. (INT 1: Pharmacist 008)*

### Shared decision making

Most professionals gave examples of trying to involve individuals in the DMR decision-making process. How much professionals were able to engage individuals and how much individuals wanted to be involved varied.

*I think the best thing is to get them to engage, often they don’t know what they’re taking. If you put some emphasis of education as well, I think they respond quite a lot. (FG2: Pharmacist 004)*

### Personability

Pharmacists presented themselves as being able to connect with DMR recipients to have meaningful conversations, which was a driver for engagement in the DMR process.

*When you go to someone’s house, you have to be respectful and you can build up bonds with them and most of the time they are quite willing. (FG2: Pharmacist 003)*

The pharmacists also suggested that at times conducting a DMR and connecting with participants can help address social isolation.

## Theme 6: outcomes

Pharmacists were asked about the outcomes they routinely capture and what they would like to capture in an ideal world to demonstrate the impact of DMRs. Outcomes were mandated by a combination of Clinical Commissioning Groups (CCGs), research bodies (regional innovation centres), and the professionals providing the service.

### Access outcomes

Focus group three highlighted a belief that their interventions had improved patients’ access to medications.

### Adherence outcomes

Most Pharmacists highlighted that they could have a positive impact on medication taking behaviours, increasing the likelihood of adherence. Examples of how this was achieved included medication-related education and provision of aids to help adherence.

### Clinical outcomes

All pharmacists described a multitude of clinical process measures and outcomes that they recorded within their services. These included: adjustments to medication taking directions, adverse drug reactions, healthcare utilisation, mortality rates, number of medications taken, measures of appropriate prescribing, frailty scores, number of interventions and the significance of interventions.

### Economic outcomes

Although not reported as frequently as clinical outcomes some professionals measured economic outcomes i.e. whether the DMR had resulted in any monetary savings to the health system.

### Humanistic outcomes

There were examples of attempting to capture data or use narrative to try and demonstrate the difference and impact DMR interventions made to the recipients of DMRs.

*I think the biggest impact had was the narratives of the patient stories (FG1: Pharmacist 001)*

### Ideal world outcomes

When asked about ideal outcomes professionals expressed a wish to know which of their interventions had been accepted by GPs, how long an individual followed a recommendation for and the lasting impact of interventions.

*One would be, which of your recommendations have been taken up and impact of those recommendations (FG2: Pharmacist 003)*

Others reported they would like to record patient satisfaction or have a greater understanding of what the changes meant to the individual receiving the DMR, something they had not found easy to do.

## Discussion

The findings of this study suggest that there is heterogeneity within DMR services; how they are set up, how DMRs are carried out, how interventions are implemented and how actions are recorded. Heterogeneity in medication review delivery is a phenomenon that has also been observed in primary care services internationally [20]. Given this heterogeneity it is important that the value of each medication review setting is explored and understood before comparisons are made between settings. This study provides an insight for the domiciliary setting.

Being able to spend more time with individuals was presented as an advantage by the pharmacists. They were able to build up rapport with individuals and conduct in-depth reviews. Participants also reported that the individuals they reviewed valued the increased time. This matches the findings of earlier patient interviews [9]. There is a suggestion that the value of DMRs can be represented by an input (time spent) rather than solely by outputs (outcomes).

Although time spent with individuals was presented as a positive, it was also acknowledged that DMRs are a time intensive service. Understanding the time taken to conduct a DMR, including any preparation and post-review actions is important. If it is recognised that DMRs take time then perhaps number of reviews is not a good measure of the value of DMRs. A focus on process measures could push emphasis to quantity rather than quality of reviews.

Comprehensiveness was also highlighted as an advantage, but it may something of a necessity given the complexity of the situations the DMRs reveal. Pharmacists have unique expertise and experience making them the best equipped professionals to conduct medication these reviews. However, this study revealed they also need to be able to recognise and take action to resolve other issues uncovered linked to the wider determinants of health. Recent studies have shown that domiciliary settings result in the identification of more MRPs [21, 22]. Being able to address the wider needs of patients during a DMR is a novel insight.

Working with other professionals either directly as part of a multi-disciplinary team, or indirectly is essential to enable DMR pharmacists to resolve the wide-ranging health and social issues they identified. Earlier research examining medication reviews of different levels, in traditional settings, also found that multi-professional input helped medication reviews be more effective [2].

Conducting DMRs expanded professional boundaries and involved tasks traditionally conducted by other professionals. Provided this is appropriate and safe, DMRs could be an example of pharmacists taking on expanded and enhanced roles in line with the NHS long-term plan [23]. The enhanced role DMR pharmacists have taken on could be contributing to the reward highlighted. The quality of the DMR interaction also enhanced the reward for pharmacists. Research has suggested that pharmacists are not motivated by renumeration but by other aspects of their role [24]. The benefits of a DMR may not only be for the recipient of the review, they can also have a wider impact on the professionals involved.

At times pharmacists reported they felt isolated. From a service planning point of view, it is important that mangers are aware of the potential drawbacks of DMRs for professionals, so that they can be addressed and avoided. Safety concerns were also highlighted as a drawback to DMRs, this study is not the only research to raise this point [25]. Taking measures to ensure the safety of DMR professionals should be a priority when services are set up. Professionals will need to feel safe to be effective in their roles. If they do not it is unlikely that they will feel comfortable spending increased amounts of time building rapport with individuals in their home, an important aspect of the DMR process.

Pharmacists reported that DMRs are rarely requested by individuals themselves. Despite this, they did not report this as a barrier to engagement. Pharmacists reported that understanding the individuals’ objectives and trying to meet them is an important part of the DMR, regardless of the origins of the DMR. The professionals also highlighted the importance of shared decision making (SDM). Either they are trying to use SDM methods in their reviews or the individual and/or carer has shown a desire to be involved with decision making. There is evidence to suggest that medication reviews can increase patient engagement with their health [26]. Evidence from Australia’s Home Medication Review (HMR) service has highlighted confusion as to the purpose of reviews as a barrier to engagement [16]. Pharmacists need to be able to engage individuals in a discussion about their priorities, which may align with the professional’s objectives after thoughtful conversation.

Clinical outcomes and adherence outcomes were the most commonly reported outcomes. Focus on clinical outcomes appears to be embedded into the psyche of pharmacy professionals [5]. Even if clinical outcomes have not been mandated by a commissioner, professionals believe they are important record them.

The pharmacists highlighted the holistic nature of DMRs but outcomes they are measuring do not support this. At times patient satisfaction is captured but nothing more in-depth. There is no check back with individuals to find out what matters to them. This is a point acknowledged by pharmacists, they want to measure patient-centric outcomes, but they do not.

## Limitations

It could be argued that the change of method from focus groups to interviews limited the richness of some of the views shared by interviewees. When the professionals were interviewed alone, they did not have others to feed off and reply to. However, the change was necessary to capture the views of professionals when schedules could not be matched to enable a focus group. Any loss of richness was limited by using a topic guide with prompts and probes to drive conversations.

As this was exploratory research focused on the DMR services rather than the individual professionals, demographic data were not collected for study participants. This limits the parallels that can be drawn to professionals providing DMR services outside of this study. Future research could explore whether there are relationships between the themes revealed in this study and the demographics of DMR professionals.

Despite focus groups being advertised widely five participants were employed by the same organisation. It could be argued that this limits the generalisability of the results. However, the pharmacists worked across different services, had varying levels of experience, and had individual viewpoints to share. Finally, sample size is a potential limitation. However, in this study data saturation was reached.

## Conclusion

To our knowledge this is the first in-depth exploration of where the value of domiciliary medication reviews lies, from the perspective of the pharmacists who conduct the reviews. It presents advantages and disadvantages of DMRs, which should be considered when service planning to ensure these services are as effective as possible.

The home setting and the time spent afford advantages over other settings. The pharmacists feel they are able to conduct more in-depth medication reviews that attempt to resolve the wide-ranging needs of patients. The relationship between the professional and the individual is also important to uncovering the objectives and needs of individuals. The importance of the personality of professionals, and their ability to engage individuals in the DMR setting highlights that training needs of DMR professionals should be reviewed and perhaps standards for competencies should be set.

The large list of ‘ideal world’ outcomes demonstrates that pharmacists feel that traditional, frequently clinical outcomes that they record do not always capture the value of DMRs. The continued use of traditional clinical and economic outcomes needs to be challenged, or at a minimum they should be presented as secondary outcomes, less important to those identified by an individual. The pharmacists conducting these reviews believe the value of the service lies in the difference they have made to the individual. Future work should explore whether outcomes can be found that describe the impact of DMR services to the individual.
